# 

**Published:** 1913-08-30

**Authors:** 


### THE PHARMACEUTICAL EXHIBITION.

The Pharmaceutical Society of Austria will hold an International Pharmaceutical Exhibition during the meeting at Vienna in September of the Congress of German Scientists and Medical Practitioners. Inquiries should be addressed to the Secretary of the Society at 31 Spitalgasse, Vienna XI.

### HANDBOOKS

FOR TRAINED WORKERS

Bound in limp cloth.

1/- net. 1/1 post free.

POCKET-GUIDE SP SERIES

Suitable size for the pocket.

1/- net. 1/1 post free.

The Works which comprise the S.P. Pocket Guide Series are intended for ready reference, consequently the aim of the Publishers has been to make them as concise and lucid as possible. Each book is written by an expert on the subject of which it treats, and the utmost reliance therefore can be placed in the information it imparts.

**Essentials of Fever Nursing.** By Lytton Maitland, M.D. (Lond.), M.B., B.S., D.P.H. (Camb.).

**Manual and Atlas of Swedish Exercises.** *(With over 60 Illustrations.)* By Thomas D. Luke, M.D., F.R.C.S.

**Bandaging Made Easy.** *(Illustrated with over 90 Diagrams.)* By M. Hosking, Sister-in-Charge, Tredegar House.

**How to Write and Read Prescriptions.** By Lytton Maitland, M.D. (Lond.), M.B., B.S., D.P.H. (Camb.).

**Principal Drugs and their Uses.** By A Pharmacist.

**Treatment after Operations.** *Rules for Nursing after General and Special Operations.* By Mary Wiles.

**The Nurse's Duties Before and During Operations.** By Margaret Fox, Matron, Prince of Wales's General Hospital, Tottenham.

**Asepsis and How to Secure It.** By H. W. Carson, F.R.C.S. *[In the Press.*

***Other Works, in amplification of this Series, will be announced in due course.***

*Published by*

THE SCIENTIFIC PRESS, Ltd.,

28/29 Southampton Street, Strand, London, W.C.

### Gifts and Bequests.

The Bristol Royal Infirmary has received a legacy of £250 from the estate of the late Mr. W. A. Lyddon. A legacy for the same amount has been received also by the Bristol General Hospital.

Mrs. Fanny Eliza Steel has bequeathed £200 each to the Sunderland Infirmary and the Sunderland Orphan Asylum; and £100 each to the Sunderland Institute for the Blind and the Sunderland Eye Infirmary.

Mrs. Agnes Tryphena O'Callaghan, of Cheltenham, has left £3,000 to the Warneford Hospital, Leamington, in memory of her three sisters; £2,000 to the Cheltenham General Hospital; £500 to the Cheltenham Home for Sick Children; £300 to the Victoria Home for District Nurses, Cheltenham; and £200 to Dr. Barnardo's Homes.

